# Discovery of a novel dehydratase of the fatty acid synthase type II critical for ketomycolic acid biosynthesis and virulence of *Mycobacterium tuberculosis*

**DOI:** 10.1038/s41598-020-58967-8

**Published:** 2020-02-07

**Authors:** Cyril Lefebvre, Wafa Frigui, Nawel Slama, Françoise Lauzeral-Vizcaino, Patricia Constant, Anne Lemassu, Tanya Parish, Nathalie Eynard, Mamadou Daffé, Roland Brosch, Annaïk Quémard

**Affiliations:** 1 Département Tuberculose & Biologie des Infections, Institut de Pharmacologie et de Biologie Structurale, UMR5089, Université de Toulouse, CNRS, UPS, 31077 Toulouse, Cedex 04, France; 2 Institut Pasteur, Unit for Integrated Mycobacterial Pathogenomics, CNRS UMR3525 Paris, France; 3 TB Discovery Research, Infectious Disease Research Institute, Seattle, WA USA; 4 Present Address: Toulouse White Biotechnology (UMS INRA / INSA / CNRS), Ramonville Saint-Agne, France; 5 Present Address: Centre de Recherches en Cancérologie de Toulouse, UMR1037 Inserm/UPS, Toulouse, France; 6 Present Address: Center for Global Infectious Disease Research, Seattle Children’s Research Institute, Seattle, WA USA

**Keywords:** Fatty acids, Bacterial physiology

## Abstract

The fatty acid synthase type II (FAS-II) multienzyme system builds the main chain of mycolic acids (MAs), important lipid pathogenicity factors of *Mycobacterium tuberculosis* (*Mtb*). Due to their original structure, the identification of the (3 *R*)-hydroxyacyl-ACP dehydratases, HadAB and HadBC, of *Mtb* FAS-II complex required in-depth work. Here, we report the discovery of a third dehydratase protein, HadD_*Mtb*_ (Rv0504c), whose gene is non-essential and sits upstream of *cmaA2* encoding a cyclopropane synthase dedicated to keto- and methoxy-MAs. *HadD*_*Mtb*_ deletion triggered a marked change in *Mtb* keto-MA content and size distribution, deeply impacting the production of full-size molecules. Furthermore, abnormal MAs, likely generated from 3-hydroxylated intermediates, accumulated. These data strongly suggest that HadD_*Mtb*_ catalyzes the 3-hydroxyacyl dehydratation step of late FAS-II elongation cycles during keto-MA biosynthesis. Phenotyping of *Mtb hadD* deletion mutant revealed the influence of HadD_*Mtb*_ on the planktonic growth, colony morphology and biofilm structuration, as well as on low temperature tolerance. Importantly, HadD_*Mtb*_ has a strong impact on *Mtb* virulence in the mouse model of infection. The effects of the lack of HadD_*Mtb*_ observed both *in vitro* and *in vivo* designate this protein as a bona fide target for the development of novel anti-TB intervention strategies.

## Introduction

Tuberculosis (TB) is the top infectious killer worldwide^1^. The control of this disease has been challenged by the emergence of multidrug and extensively drug-resistant *Mycobacterium tuberculosis* (*Mtb*) strains. According to the WHO, the development of a new generation of drugs effective against these strains is urgently needed^1^. The very thick lipid-rich envelope of the tubercle bacillus provides a vital protective coat against the attacks of the infected host. In particular, the mycolic acid (MA)-containing lipids, which play a strategic role in the envelope architecture and permeability, are essential to the mycobacterial survival and constitute important pathogenicity factors. As a consequence, their biosynthesis pathway represents one of the Achilles’ heels of the tubercle bacillus. It is the primary target of several anti-TB antibiotics, including the first line drug isoniazid^2,3^. Furthermore, recently discovered small molecules, that are showing great promise as TB therapeutics, affect their metabolism^4^.

MAs, extremely long-chain *α*-alkylated *β*-hydroxylated fatty acids (FAs), are the major components of a highly efficient permeability barrier, the mycobacterial outer membrane (called mycomembrane)^5^, where they are covalently linked to the arabinogalactan layer or to polyol molecules such as trehalose^6^. Their production requires three distinct multienzyme systems, including the acyl carrier protein (ACP)-dependent fatty acid synthase type II (FAS-II) that is responsible for the synthesis of their main chain called ‘meromycolic chain’^6^. The latter carries various types of chemical function (cyclopropane ring, double bond, methyl branch and oxygenated groups) that differentiate the MA subclasses and modulate their biological activities. While typical FAS-II systems found in plants, bacteria, parasites, and mitochondria perform *de novo* biosynthesis^7^, the system from the *Corynebacteriales* order, which includes the *Mycobacterium* genus, elongates standard-size FAs (C_16_–C_18_)^8^. This unique property explains the success of the mycobacterial FAS-II system as a target for specific anti-TB drug therapy, illustrated by the modes of action of the drugs isoniazid, ethionamide and thiacetazone^2^. In mycobacteria, enzymes catalyzing the four main elongation steps have been characterized^9–13^. The last enzymes identified in *Mtb* FAS-II were two heterodimeric (3*R*)-hydroxyacyl-ACP dehydratases (HADs), HadAB and HadBC^13^. Belonging to the hydratase 2 family, they have an original structure for HADs of FAS-II system^13–15^, which are classically FabZ/FabA-type proteins.

In a recent survey, a combination of pull-downs, using the dehydratase HadAB of FAS-II as a bait, and proteomic analyses led to the discovery of a novel (*R*)-specific dehydratase, HadD_*Msm*_, of the FAS-II system from *M. smegmatis*, a non-tuberculous mycobacterium^16^. HadD displays highly specific interactions with HadAB. Bioinformatic analyses showed that, unlike HadAB, there is no HadD_*Msm*_ ortholog in the genera of the *Corynebacteriales* order, such as *Nocardia*, *Rhodococcus*, and *Gordonia*, that produce medium-chain mycolic acids^16^. This suggested that HadD_*Msm*_ might have a role during the late FAS-II elongation cycles. Consistent with this, the deletion of *hadD*_*Msm*_ (*MSMEG_0948*) gene quasi-totally abolished the biosynthesis of the long chain α- and epoxy-MAs, and the resulting mutant strain produced only the medium-size α’-MAs. Thus, HadD_*Msm*_ is most likely involved in building the third meromycolic segment leading to the synthesis of the full-size α- and epoxy-MAs. Importantly, *hadD*_*Msm*_ inactivation induced an upheaval of both the bacterial cell surface and envelope properties of *M*. *smegmatis*, strongly altering the bacterial fitness and capacities to aggregate, assemble into colonies or biofilms and spread by sliding motility, critical for mycobacterial survival under stress and hostile conditions and for colonization^16^. It also conferred a hypersensitivity to the first line TB drug rifampicin.

Interestingly, a putative ortholog of HadD_*Msm*_ was found in all of the sequenced mycobacterial genomes^16^, including those of the pathogen *Mtb*. Yet, *Mtb* produces a MA combination distinct from that of *M. smegmatis*, *i.e*. α-, keto- and methoxy-MAs, and has no α’- and epoxy-MAs^2^. In this context, we sought to elucidate the role of the putative ortholog of HadD_*Msm*_ in *Mtb*, which in the reference strain H37Rv is represented by the yet uncharacterized protein Rv0504c. Furthermore, given the importance of HadD_*Msm*_ for *M. smegmatis* physiology, we generated a *Rv0504c* knock-out *Mtb* mutant and examined the impact of *Rv0504c* inactivation on different physiological properties of *Mtb* in axenic cultures as well as on its virulence in the mouse model of infection.

## Results

### *M. tuberculosis* holds a putative HadD ortholog that is not essential for survival

**Figure 1:**
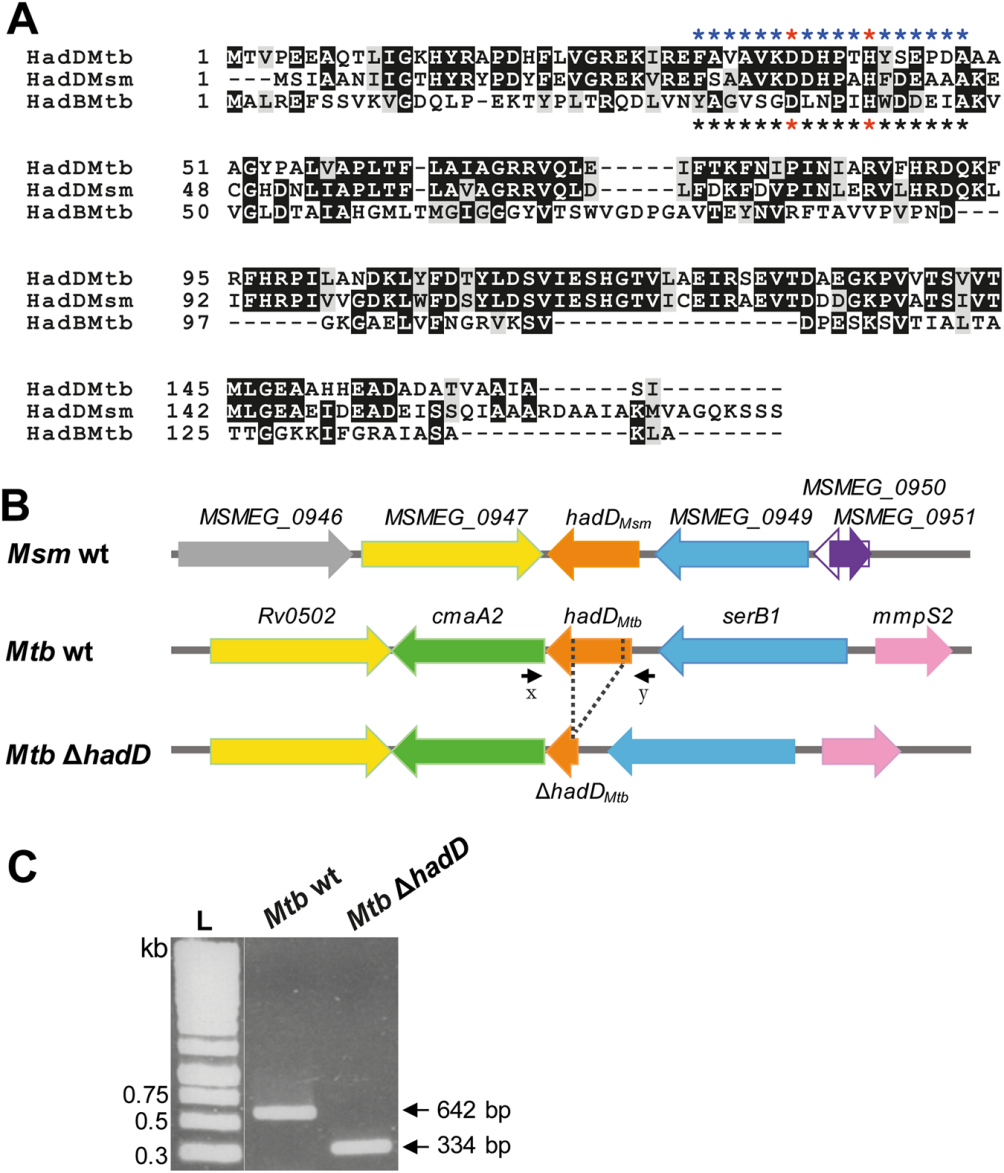
Analysis of HadD_*Mtb*_ sequence, *hadD* chromosomic region and *Mtb* ∆*hadD* mutant. (**A**) Sequence alignment of HadD_*Mtb*_ (Rv0504c) with HadD_*Msm*_ (MSMEG_0948) and HadB_*Mtb*_ (Rv0636) proteins. Black and gray shadings indicate strictly conserved and similar residues, respectively. HadD_*Mtb*_ shares a sequence identity of 63% with HadD_*Msm*_ (68% using BlastP alignment) and only 19% with HadB_*Mtb*_ (Clustal Omega scores). HadD_*Mtb*_ bears a degenerate hydratase 2 motif ‘F-x(2)-a-x(2)-**D**-x(2)-P-x-**H**-x(5)-A’ (uppercase: strictly conserved; lowercase: similar residue) indicated by blue stars; the putative catalytic Asp and His residues are labeled by red stars. The hydratase 2 motif ‘[YF]-x(1,2)-[LIVG]-[STGC]-G-D-x-N-P-[LIV]-H-x(5)-[AS]’ of HadB_*Mtb*_^13^ is indicated by black stars. Alignment was performed by using Clustal Omega program, and the figure was shaped with Box shade. Database accession numbers: HadD_*Mtb*_, P9WFK3 (166 aa); HadD_*Msm*_, A0QR13 (177 aa); HadB_*Mtb*_, I6WYY7 (142 aa). (**B**) Genomic organization of *hadD* gene region in *M. smegmatis, Mtb* H37Rv and *Mtb* Δ*hadD* strains. Matching genes are drawn with identical colors. *Mtb* Δ*hadD* mutant strain was produced by an in-frame deletion of a 308 bp internal fragment (dashed lines) of *hadD*_*Mtb*_ gene (501 bp). In *Mtb*, *Rv0502* (1,077 bp), *cmaA2* (909 bp), *serB1* (1,122 bp) and *mmpS2* (444 bp) are annotated as encoding a conserved protein, the mycolic acid cyclopropane synthetase CmaA2, a possible phosphoserine phosphatase SerB1 and a probable conserved membrane protein MmpS2, respectively. The distinct genes in *M. smegmatis*, *MSMEG_0946*, *MSMEG_0950* and *MSMEG_0951*, are annotated as encoding a NAD-dependent epimerase/dehydratase family protein, a hypothetical protein and the glutaredoxin 2, respectively. (**C**) Verification of *hadD*_*Mtb*_ gene deletion by PCR analysis. The primers (x and y; symbolized by black arrows in panel B) used for the PCR are located outside the *hadD*_*Mtb*_ gene. The genomic DNA of each strain was used as a template. *HadD*_*Mtb*_ gene length: 501 bp; Δ*hadD*_*Mtb*_ gene length: 193 bp. L: DNA ladder.

Protein–protein BLAST searches performed against *Mtb* H37Rv genome^17^, using the MSMEG_0948 (HadD_*Msm*_) protein sequence as a probe, showed the presence of a potential ortholog of HadD_*Msm*_ with a sequence identity rate of 68% in *Mtb*, Rv0504c, which we named HadD_*Mtb*_ (Fig. 1A). The latter, annotated as “conserved protein” and having a theoretical monomeric mass of 18.4 kDa^17^, bears similarly to HadD_*Msm*_ a degenerate hydratase 2 motif ‘F-x(2)-a-x(2)-**D**-x(2)-P-x-**H**-x(5)-A’ containing the putative catalytic dyad Asp (D37) and His (H42) (Fig. 1A). The chromosomal region of *hadD* gene is partially conserved between *Mtb* and *M. smegmatis* (Fig. 1B). Interestingly, *cmaA2* (*Rv0503c*) gene sits downstream of *hadD*_*Mtb*_ (*Rv0504c*) on *Mtb* chromosome and is transcribed in the same direction. It encodes the mycolic acid methyltransferase (MA-MT) CmaA2 that has a function of cyclopropane synthase and introduces a *trans* or *cis* cyclopropane at the proximal position of both keto- and methoxy-MAs^18^. The lack of a *cmaA2* ortholog in *M. smegmatis* (Fig. 1B) is in agreement with the absence of these MA classes in this species^2^.

The essentiality of *hadD*_*Mtb*_ gene was examined by generating an in-frame unmarked deletion (Fig. 1B) using a two-step homologous recombination method^19^, so that it does not cause any polar effect on *cmaA2* expression. The gene deletion was verified by PCR analysis (Fig. 1C). The viability of the resulting mutant strain showed that *hadD*_*Mtb*_ is not essential for the survival of *Mtb* in axenic culture, similar to the situation seen for *hadD*_*Msm*_ in *M. smegmatis*^16^. This is consistent with the prediction of non-essentiality of *hadD*_*Mtb*_ made from a microarray-based study^20^, but in discrepancy with a more recent survey using high-resolution phenotypic profiling where *hadD*_*Mtb*_ was predicted to be essential^21^.

### *HadD*_*Mtb*_ deletion alters *Mtb* physiology and virulence

**Figure 2:**
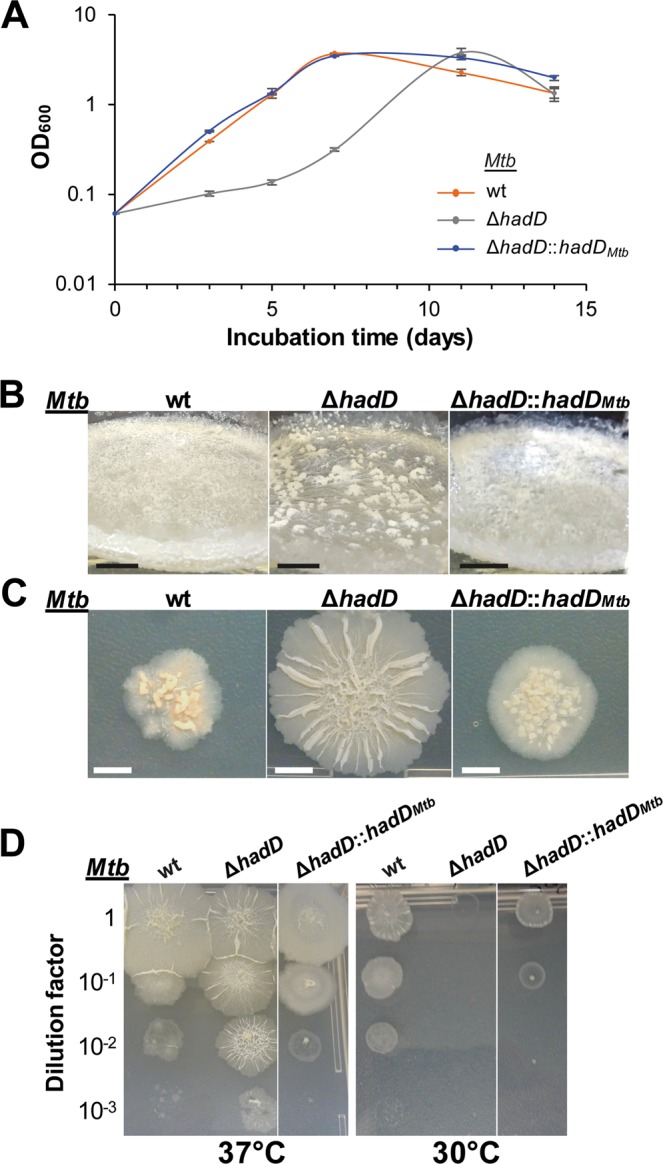
Inactivation of *hadD*_*Mtb*_ alters the bacterial fitness, biofilm and colony formation, and tolerance to low temperature. Comparison of *Mtb* H37Rv wt, Δ*hadD* and the complemented Δ*hadD*::*hadD*_*Mtb*_ strains in different phenotyping assays. All of the data are representative of at least three independent experiments. (**A**) Planktonic growth. Cultures were performed under shaking (120 rpm) at 37 °C in 7H9-based medium supplemented with 0.05% (w/v) Tween-80. Data are means and average deviations of three independent experiments. Some deviation bars are too small to be visible. (**B**) Biofilm growth at the air-liquid interface. The growth was followed for three weeks at 37 °C on 7H9-based medium. Photographs were taken after three weeks. Scale bars represent 1 cm. (**C**) Colony morphotype. Five μl culture aliquots were spotted on 7H11-based medium and grown for three weeks at 37 °C. Scale bars represent 0.5 cm. (**D**) Sensitivity to low temperature. Liquid precultures were adjusted to the same OD then serially diluted, spotted onto 7H11-based medium and incubated for four weeks at 37 °C or 30 °C.

In the aim of evaluating the importance of HadD_*Mtb*_ function in the physiology of *Mtb*, phenotypic assays were realized. Although *Mtb* Δ*hadD* is viable, this mutant strain showed difficulties to grow in planktonic culture as compared to the wild type (wt) strain (Fig. 2A). *HadD*_*Mtb*_ inactivation also resulted in a strong alteration of the structuration of biofilms at the air-liquid interface, which appeared thinner with large clumps (Fig. 2B). There was a deep change of the colony morphology as well, with much larger, flat and spread colonies (Fig. 2C). The wt phenotype was restored in the mutant upon complementation meaning that these phenomena were linked to *hadD*_*Mtb*_ deletion. The lack of HadD_*Mtb*_ also conferred a high sensitivity to low temperature (Fig. 2D). In addition, *Mtb* Δ*hadD* was slightly more susceptible than *Mtb* wt to rifampicin, a first line antituberculous drug targeting the DNA-dependent RNA synthesis. In contrast, there was no significant difference in the sensitivity of both strains to isoniazid and ethambutol, two other firstline TB drugs, and to ciprofloxacin, a broad spectrum fluoroquinolone, as well as to the SDS detergent (Supplementary Table S1).

**Figure 3:**
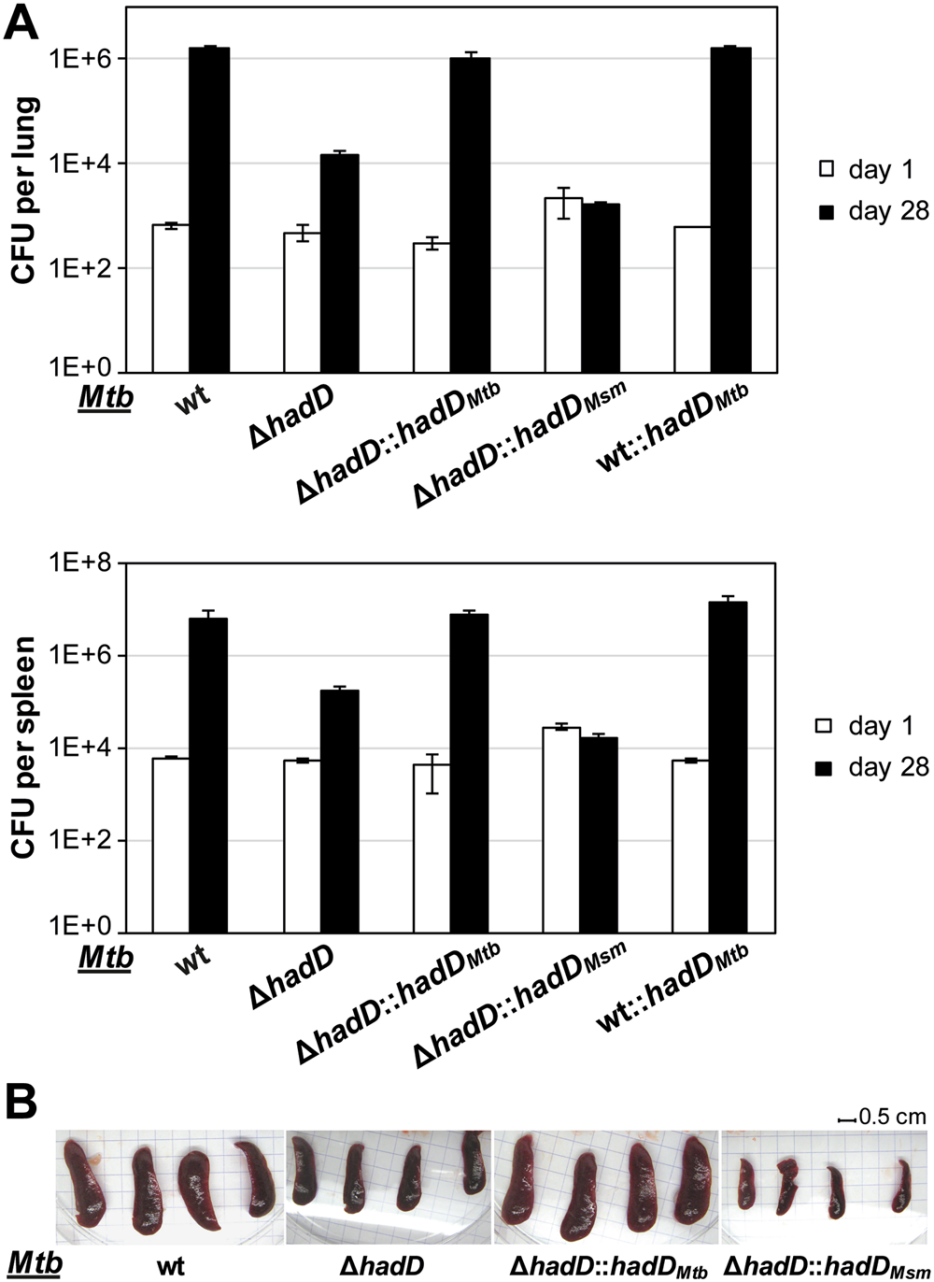
The deletion of *hadD* strongly affects *Mtb* virulence. The *Mtb* ∆*hadD* mutant had a reduced capacity to grow in the lungs and spleen of SCID mice. Mice were infected intravenously with 1–2 × 10^5^ CFU of the different *Mtb* wt and mutant strains. (**A**) Bacterial loads in lungs (top panel) and spleens (bottom panel) determined after 1 (white bars) and 28 days (black bars). Data reported are mean and standard deviation of values obtained in a representative experiment of three performed each with four mice per group. (**B**) Photographs of the spleens from infected mice at 28 days post-infection.

To assess the influence of *hadD*_*Mtb*_ on the virulence level of *Mtb*, infection trials were performed with severe combined immunodeficiency (SCID) mice. This murine model provides a rapid and sensitive method for evaluating *in vivo* growth characteristics of mycobacterial strains during the acute phase of infection^22–25^. Although overexpressing *hadD*_*Mtb*_ gene had no significant impact on the virulence level, we observed marked and reproducible reductions of the bacterial loads in lungs (2 logs) and spleens of mice infected with the deletion mutant after 28 days of infection relative to controls (Fig. 3A). This effect correlated with a decrease of the spleen size (Fig. 3B). This phenomenon likely reflected a specific *in vivo* phenotype since the bacterial density of *Mtb* ∆*hadD* reached that of the wt strain after 11 days of growth in axenic cultures (Fig. 2A). The complementation observed upon transformation of the mutant strain with a wt *hadD*_*Mtb*_ copy provides explicit evidence for a link between HadD_*Mtb*_ function and *Mtb* virulence (Fig. 3A).

In conclusion, HadD_*Mtb*_ plays a key role in the faculty of the tubercle bacillus to invade and multiply within the infected host. Furthermore, it is also important for the fitness and the capacities of *Mtb* bacilli to assemble into colonies or biofilms, and for their tolerance to low temperature. The deep changes observed in *hadD*_*Mtb*_-deficient strain likely are the consequences of an alteration of the cell envelope composition and architecture.

### HadD_*Mtb*_ influences the mycolic acid profile of *Mtb*

**Figure 4:**
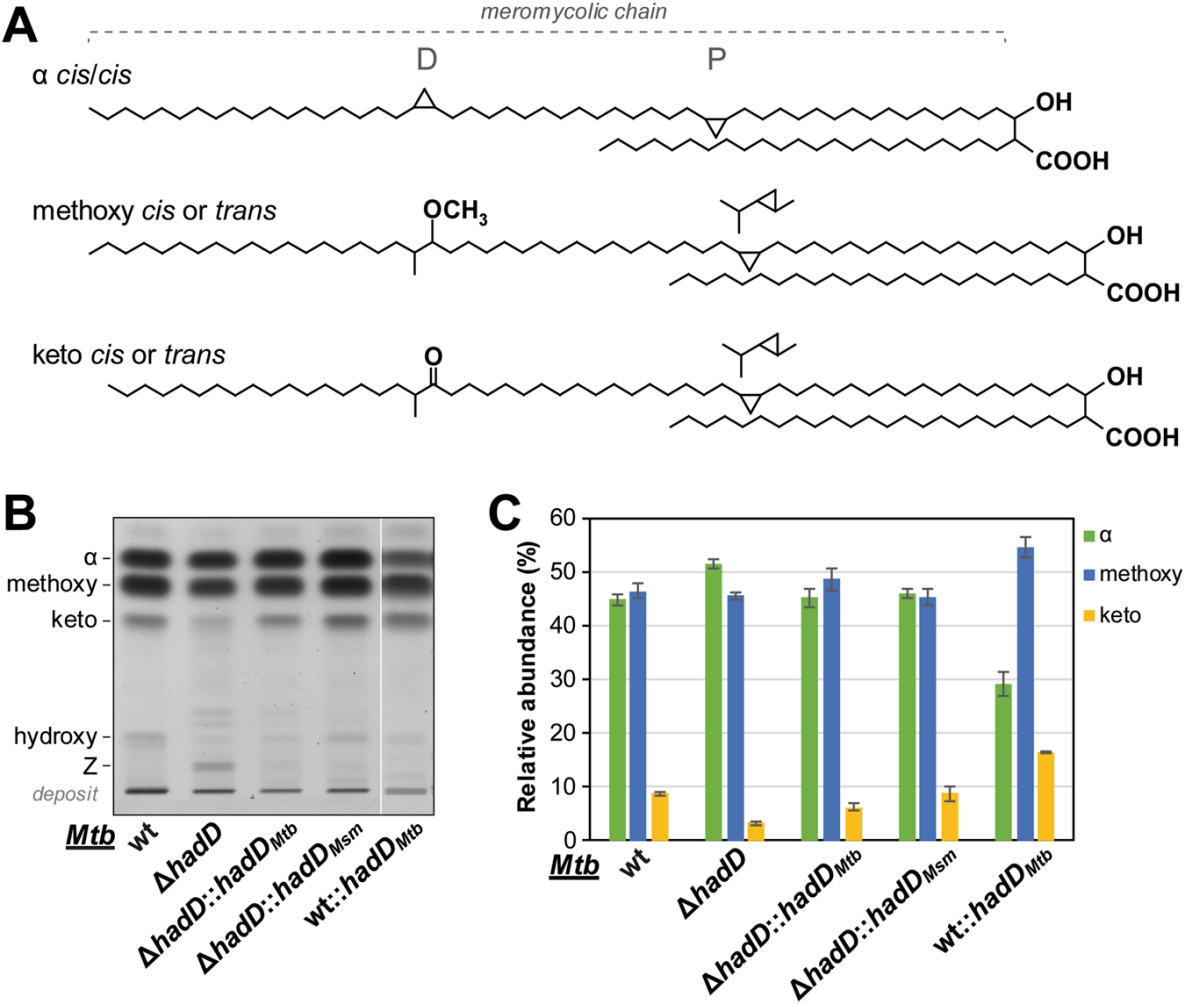
HadD_*Mtb*_ protein is implicated in keto-mycolic acid biosynthesis pathway. (**A**) Structures of the main MA classes produced by *M. tuberculosis*. One of the main molecules was drawn for each class, as an example. The three segments of the meromycolic chain, delimited by the distal (D) and the proximal (P) positions of the chemical functions, are indicated. (**B**) Mycolic acid methyl ester (MAME) profiles in HPTLC. Five µg of MAMEs from each strain were loaded onto a HPTLC plate developed five times in petroleum ether/diethyl ether, 9:1 (v/v) and stained by immersion in CuSO_4_ and heating. The figure is representative of six independent experiments. The hydroxy-MAs are minority MAs bearing an hydroxyl group at the distal position, and precursors of the keto- and methoxy-MAs^28^. (**C**) MA distribution in the different strains deduced from the quantification of the HPTLC band intensities as in panel B. Data are means ± average deviations of at least six independent experiments.

*Mtb* produces three main MA classes, α-, methoxy- and keto-MAs (Fig. 4A). To examine the potential involvement of HadD_*Mtb*_ protein in their biosynthesis, the MAs were extracted from *Mtb* ∆*hadD*_*Mtb*_ strain and analyzed. The MA content, expressed as the ratio ‘MA dry weight/delipidated bacterial residue dry weight’, was similar between the wt (10.2 ± 1.1%) and the mutant (10.4 ± 1.7%) strains. Yet, the MA distribution was changed in the deletion mutant, as shown by HPTLC analysis of the MA methyl esters (MAMEs) (Fig. 4B,C). The abundance of keto-MAs was reduced by 63%. This was compensated by an increase in the α-MA content. The recovery of the wt profile upon complementation with a wt *hadD*_*Mtb*_ copy suggested that this gene is directly involved in keto-MA biosynthesis^26^. The overexpression of *hadD*_*Mtb*_ in *Mtb* wt strain supported this conclusion, since it induced a strong increase (of 87%) in the keto-MA relative content (Fig. 4C). This had repercussions on the relative abundance of the biosynthetically affiliated methoxy-MAs, which increased slightly (Fig. 4C).

These results altogether clearly demonstrate that HadD_*Mtb*_ plays an important role in the biosynthesis of the keto-MAs. It is noteworthy that no major change in the profile of other lipids from *Mtb* was detected (Supplementary Fig. S1) suggesting that *hadD*_*Mtb*_ is not involved in another lipid biosynthesis pathways. Therefore, the significant alteration of MA distribution in *Mtb* ∆*hadD* is most likely responsible for its loss of virulence (Fig. 3A).

### Production of full-size keto-mycolic acids requires an active HadD_*Mtb*_ protein

**Figure 5:**
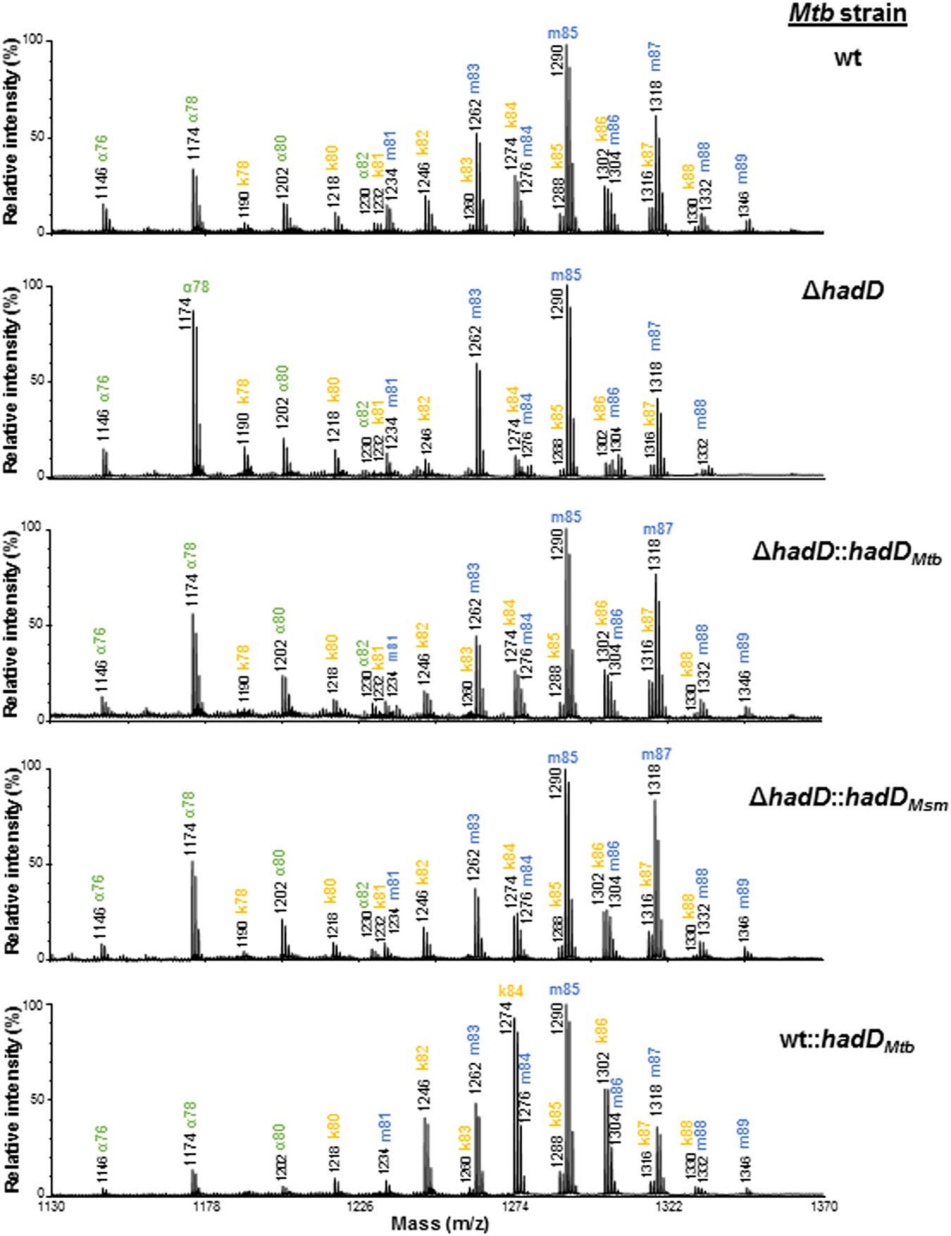
HadD_*Mtb*_ is involved in the late steps of keto-MA biosynthesis. MALDI-TOF MS spectra of the MAME mixtures from the different *Mtb* strains. Ion peaks are labeled with the matching MA type and the total carbon number (of the free acid form). They correspond to monosodium adducts. The spectra are representative of three independent experiments. k, keto-MA; m, methoxy-MA.

To investigate further the function of HadD_*Mtb*_, the fine structures of MAs were analyzed using MALDI-TOF mass spectrometry (MS) and ^1^H-NMR spectroscopy. The mass spectrum of the total MA mixture from *Mtb* ∆*hadD* displayed an increase of the relative intensities of α-MAMEs signals with respect to the wt strain (Fig. 5). More importantly, the size distribution of the keto-MAs changed significantly. The signal intensities of the long chain molecules (C_82_-C_88_) markedly decreased, while those of the short-chain keto-MAs (C_78_, C_80_) raised (Fig. 5). The same observations were made on the spectrum of keto-MAs purified from the deletion mutant (Supplementary Fig. S2). The wt profiles were partially recovered in the complemented strain *Mtb* Δ*hadD*::*hadD*_*Mtb*_ (Fig. 5, Supplementary Fig. S2), confirming the role played by *hadD*_*Mtb*_ deletion in this phenotype. It is noteworthy that a reduction in the proportion of the longest molecules was also detected for the methoxy-MAs in *Mtb* ∆*hadD* (Fig. 5). Moreover, when *hadD*_*Mtb*_ was overexpressed in *Mtb* wt, the relative content of the long-chain keto-MAs (C_82_-C_86_) strongly rised (Fig. 5).

Consistent with HPTLC analyses (Fig. 4B,C), these data altogether confirm the involvement of HadD_*Mtb*_ in the keto-MA pathway. Furthermore, they show that HadD_*Mtb*_ is implicated in the late elongation cycles during the biosynthesis of the longest chain keto-meromycolic chains. This is in agreement with the lack of HadD ortholog in other genera of the *Corynebacteriales* order, such as *Nocardia*, *Rhodococcus*, and *Gordonia*, which produce medium-chain MAs^16^. Despite a slight difference in the *cis*/*trans* cyclopropane distribution in the keto-MAs, there was no change in the type of unsaturations carried by the MAs in *Mtb* ∆*hadD*, which remained cyclopropanes (Supplementary Table S2), whereas inactivation of *cmaA2* leads to accumulation of ethylenic oxygenated MAs in *Mtb*^18^. Thus, as expected, the in-frame unmarked *hadD*_*Mtb*_ deletion performed had no visible polar effect on the expression of the adjacent *cmaA2* gene (Fig. 1B) encoding CmaA2 cyclopropane synthase.

### HadD_*Mtb*_ and HadD_*Msm*_ have distinct substrate specificities

Interestingly, HPTLC analysis showed that *hadD*_*Msm*_ gene from *M. smegmatis* expressed in *Mtb* ∆*hadD* could restore a MA distribution similar to that of *Mtb* wt strain (Fig. 4B). Furthermore, the fine structures of the MAs in *Mtb* Δ*hadD*::*hadD*_*Msm*_ were also largely recovered, as observed by MALDI-TOF MS (Fig. 5) and NMR spectroscopy (Supplementary Table S2). This suggested that the functions of HadD_*Mtb*_ and HadD_*Msm*_ proteins are similar. Indeed, the present work and previous data^16^ show that they are both involved in dehydratation steps during late FAS-II elongation cycles. Yet, unlike *hadD*_*Mtb*_, the *hadD*_*Msm*_ gene was not able to restore the virulence level of *Mtb* wt in *Mtb* Δ*hadD* strain (Fig. 3A,B). Furthermore, the reverse cross-complementation, *i.e*. the expression of *hadD*_*Mtb*_ in *M. smegmatis* ∆*hadD*, was far to be as successful in terms of MA profile and physiology. The MA distribution was much closer to that of *M. smegmatis* ∆*hadD* than that of *M. smegmatis* ∆*hadD*::*hadD*_*Msm*_ (Supplementary Fig. S3A,B). The same conclusions were drawn from the colony morphology observation (Supplementary Fig. S3C) as well as from the sensitivity assays to rifampicin (Supplementary Fig. S3D), towards which *M. smegmatis* ∆*hadD* exhibits a hypersensitivity^16^. These data show that the functions of HadD_*Mtb*_ and HadD_*Msm*_ are not completely superimposable. This is in perfect agreement with the specificities of HadD_*Msm*_ for the α- and epoxy-MA biosynthesis^16^ and of HadD_*Mtb*_ for the keto-MA pathway. The inactivation of *hadD*_*Mtb*_ did not induce a reduction of α-MA content in *Mtb* as in *M. smegmatis*^16^. Therefore, although HadD_*Mtb*_ and HadD_*Msm*_ possess similar functions, they are not strict orthologs, and the substrate specificity of HadD_*Mtb*_ seems important for its role in virulence.

### Accumulation of dehydratation substrates triggered by *hadD*_*Mtb*_ inactivation

It is noteworthy that *Mtb* ∆*hadD* accumulated a polar compound ‘Z’, undetectable in *Mtb* wt and hardly visible in *Mtb* ∆*hadD*::*hadD*_*Mtb*_ by HPTLC (Fig. 4B). This compound, appearing as a double band, likely corresponded to a heterogeneous mixture of molecules. After purification, the MALDI-TOF mass spectrum of compound Z displayed peak envelopes within a mass range from 1148 to 1362 Da (Supplementary Fig. S4A) reminiscent of the classical MAME mixture (Fig. 5), but with an addition of 16 mass units. Consistent with this, the mass increment of 84 Da (corresponding to two acetyl groups) observed after per-*O*-acetylation of compound Z indicated the presence of two hydroxyl groups in the intact compound (Supplementary Fig. S4A), which explained its low *R*_f_ in HPTLC (Fig. 4B). This was confirmed by ^1^H-NMR spectroscopy of intact compound Z generating two signals at 3.85 ppm and 3.98 ppm assigned to methines bearing both hydroxyl groups (-C**H**OH-). After peracetylation of compound Z, these signals shifted at 4.92 ppm and 5.13 ppm, corresponding to the chemical shifts of methines bearing *O*-acetyl groups (Supplementary Fig. S4B). The spin system in the ^1^H-^1^H 2D NMR COSY spectrum of per-*O*-acetylated compound Z revealed that, in the intact compound, one hydroxyl group is carried by the C-3 like in the classical MAs, whereas the additional hydroxyl group is located on the C-5 (Supplementary Fig. S4C,D). These data altogether clearly showed that compound Z is a mixture of 5-hydroxylated MAs. These molecules are mostly *cis*-cyclopropanated like regular *Mtb* MAs (Supplementary Table S2).

**Figure 6:**
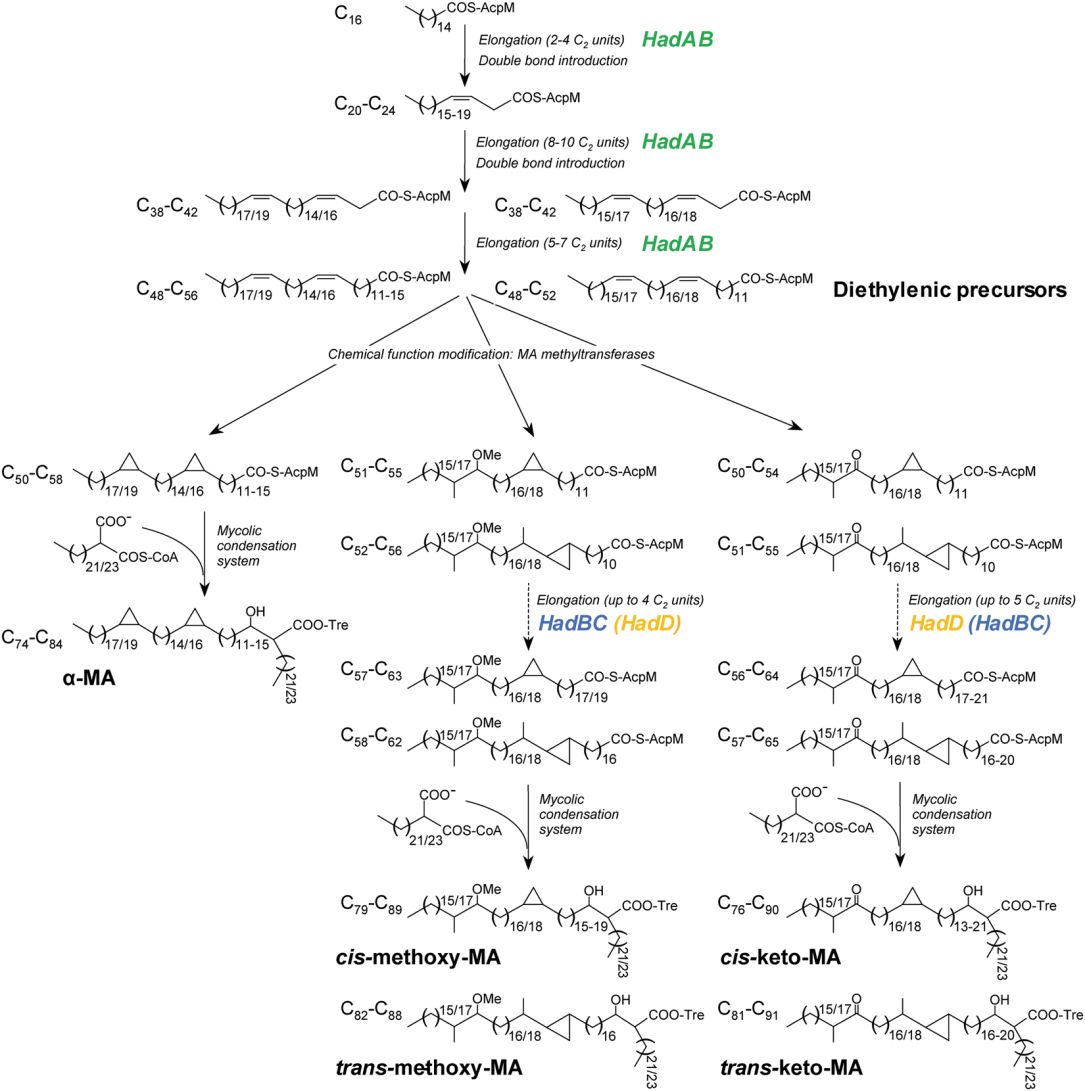
Proposed function of HadD_*Mtb*_ in the mycolic acid biosynthetic pathway of *M. tuberculosis*. FAS-II first catalyzes several series of early elongation cycles involving HadAB dehydratase, and punctuated by the introduction of two double bonds potentially leading to the biosynthesis of diethylenic precursors. These double bonds are modified into the appropriate chemical functions (*cis*/*trans* cyclopropane, methoxy, keto) by dedicated MA-methyltransferases that differentiate the pathways of the three main MA classes. The Claisen-type condensation of the resulting dicyclopropyl meromycoloyl chains with a carboxy-fatty acyl-CoA by the mycolic condensation system generates the trehalose α-MA esters. The formation of the main methoxy- and keto-MAs, whose meromycolic chain is longer, requires an additional set of FAS-II late elongation cycles, which specifically involve HadBC and HadD_*Mtb*_ dehydratases during the methoxy-MA and keto-MA biosyntheses, respectively. HadBC and HadD_*Mtb*_ functions would partially overlap. The corresponding mature MAs are then generated by the mycolic condensation system. The total carbon number of the meromycolic and MA chains are indicated. AcpM, mycobacterial FAS-II acyl carrier protein.

The additional hydroxyl group on the C-5 in compound Z was located on the C-3 in the precursor meromycolic chains, before the mycolic condensation step (Supplementary Fig. S5). The 3-hydroxylated meromycolic acids were taken over by the mycolic condensation system leading to the synthesis of abnormal 5-hydroxylated MAs. The accumulation of 3-hydroxylated intermediates corresponding to dehydratase substrates strongly suggests that, in the absence of HadD_*Mtb*_ protein, the dehydratation step of the late FAS-II elongation cycles is partially blocked, preventing the formation of mature meromycolic chains (Supplementary Fig. S5). The 3-hydroxylated α-meromycolic chains might come from the group of diethylenic precursors of oxygenated MAs (Fig. 6), which, accumulating abnormally, would later be cyclopropanated by default like the regular α-meromycolic chains.

In conclusion, HadD_*Mtb*_ most likely catalyzes the 3-hydroxyacyl-ACP dehydratation step of the late elongation cycles during the biosynthesis of the oxygenated meromycolic chains.

## Discussion

The present study reports the existence in *Mtb* of a putative ortholog, which we named HadD_*Mtb*_, of the recently discovered (3*R*)-hydroxyacyl-ACP dehydratase HadD_*Msm*_ of the FAS-II system from *M. smegmatis*^16^. The in-depth analysis of the distribution and fine structure of the MAs produced by *Mtb hadD* deletion mutant revealed that HadD_*Mtb*_ is involved in the oxygenated MA biosynthesis, and more particularly dedicated to the keto-MA pathway. Indeed, in the mutant, the overall keto-MA content was strongly reduced. The fact that the relative abundance of the longest keto-MAs decreased in *Mtb* ∆*hadD*_*Mtb*_ whereas it increased in *Mtb* wt::*hadD*_*Mtb*_ overexpression strain shows that HadD_*Mtb*_ is involved in late FAS-II elongation cycles during the biosynthesis of the keto-meromycolic chains. This conclusion is supported by the lack of HadD ortholog in the genera of the *Corynebacteriales* order that produce only intermediate size and not full size mycolic acids as found in mycobacteria^16^. The abnormal 5-hydroxylated MAs observed in HadD_*Mtb*_ deficient strain result from the condensation of 3-hydroxy-meromycoloyl chains with a carboxyacyl chain (Supplementary Fig. S5). Their accumulation together with the belonging of HadD_*Mtb*_ to the hydratase 2 protein family and its high sequence similarity with HadD_*Msm*_ leads to the conclusion that HadD_*Mtb*_ most likely catalyzes the 3-hydroxyacyl-ACP dehydratation step of these FAS-II elongation cycles (Fig. 6). We had previously discovered two (3 *R*)-hydroxyacyl-ACP dehydratases of the FAS-II system from *M. tuberculosis*, HadAB and HadBC^13^. Thus, HadD_*Mtb*_ constitutes a third dehydratase of this system. It has been shown that HadAB is involved in the early elongation cycles common to both α and oxygenated MA pathways, while HadBC is required for the late cycles leading to the biosynthesis of the sole oxygenated MAs, which are 4–6 carbon longer than α-MAs^13,27^. Since the keto- and methoxy-MAs are biosynthetically affiliated^28^, the MA distribution in *Mtb* ∆*hadC* strain was interpreted in terms of α/oxygenated ratio^27^. Yet, it is noteworthy that, in this mutant, the keto-MA content remains stable whereas that of the methoxy-MAs drops dramatically^27^. In the light of our new findings, this strongly suggests that HadBC is preferentially dedicated to the methoxy-MA biosynthesis and HadD_*Mtb*_ to the keto-MA pathway. However, the functions of both enzymes are likely partially redundant since their individual inactivation does not totally inhibit the production of methoxy- or keto-MAs. This is supported by the variations in methoxy-MA content and size distribution observed in *Mtb* wt::*hadD*_*Mtb*_ and in *Mtb* ∆*hadD*_*Mtb*_, respectively (Figs. 4 and 5), and in keto-MA fine structure in *Mtb* Δ*hadC* strain^27^, which may also be partly due to a tight regulation between both biosynthesis pathways. These surveys allow us to draw a biosynthesis scheme for the three MA classes from *Mtb*, which details the specific roles of the three FAS-II dehydratases (Fig. 6).

The functions of HadD_*Mtb*_ and HadD_*Msm*_ in *M. smegmatis* appear closely related since they both catalyze the dehydratation step during late elongation cycles^16^. Yet, the protein from *Mtb* has a substrate specificity distinct from that of *M. smegmatis*, where it is required for the biosynthesis of α- and epoxy-MAs^16^. The phenotypic analyses of the cross-complemented *M. smegmatis* ∆*hadD* mutant in terms of MA profile, colony morphology and rifampicin sensitivity, and of the cross-complemented *Mtb* ∆*hadD* mutant in terms of virulence level confirmed this conclusion and definitely showed that *hadD*_*Mtb*_ and *hadD*_*Msm*_ are not strict orthologs, despite the partial genomic conservation of their chromosomal regions. This suggests that there has been a functional divergence of both genes after the speciation event. Indeed, during its adaption to a distinct environment, the ortholog in a new species may undergo neofunctionalization, resulting in a species-specific function for this gene^29^.

We showed that *Rv0504c* gene encoding HadD_*Mtb*_ is not essential for the survival of the tubercle bacillus in axenic culture. However, we observed that it has an important impact on the fitness of bacteria, their organization into biofilms and colonies, as well as their tolerance to low temperature (30 °C). This is certainly linked to the determinant role of MAs in the architecture and the fluidity of the envelope due to their strategic location within the mycomembrane^2,30^. In agreement with our data, the growth of an *Mtb* strain overproducting *mmaA3* MA-methyltransferase gene and lacking keto-MAs was impaired at reduced temperature (32 °C)^31^. MAs are also known to be important for the formation of mycobacterial biofilms, where the extracellular matrix contains large amounts of free MAs^32^. In particular, the production of keto-MAs in which HadD_*Mtb*_ is involved is essential for biofilm growth^33^. MAs represent also key *Mtb* pathogenicity factors, their fine structures strongly potentiating the immune response to infection^2^. Here, we show that HadD_*Mtb*_ function greatly influences the virulence level of *Mtb* in the mouse model of infection. This is consistent with previous findings showing that a *Mtb hma* (*mmaA4*) mutant devoid of oxygenated MAs is attenuated in mice^34^ and that keto-MAs are critical for *Mtb* growth within the natural host cells^31^. Additionally, oxygenated MAs play a role in the selective repression of macrophage IL-12p40 cytokine production aimed to evade elimination by the host immune system^35^, and the *hma* gene required for their biosynthesis is actively expressed during human pulmonary tuberculosis^36^. Importantly, the recently approved drug delamanid, active against MDR-TB, kills *Mtb* by blocking the oxygenated MA production^37^. Altogether, these data indicate that targeting this metabolism would constitute a relevant strategy for the development of new therapeutics against *Mtb*. Because of its involvement in the keto-MA biosynthesis pathway, its role in *Mtb* virulence and its specificity to mycobacterial cells^16^, HadD protein represents a promising pharmaceutical target. Besides, targeting non-essential enzymes of the tubercle bacillus should limit the occurrence of antibiotic resistance mechanisms.

## Methods

### Bio-containment measures

All of the experiments using *Mtb* living strains were performed either in a regular Biosafety Level-3 (BSL-3) laboratory or in an Animal Biosafety Level-3 (ABSL-3) laboratory in the case of mouse infection experiments.

### Protein sequence analyses

Sequence alignments were performed using BLAST and Clustal Omega softwares^38,39^, with default parameters. The presence of a putative HadD_*Msm*_ ortholog in *Mtb* H37Rv was analyzed by BlastP searches against the fully sequenced genome available at NCBI website (www.ncbi.nlm.nih.gov/genomes/lproks.cgi), using MSMEG_0948 protein sequence (database accession number: A0QR13**)** as a probe. Genomic organization of *hadD*_*Msm*_ and *hadD*_*Mtb*_ gene regions in *M. smegmatis* mc^2^155 and *Mtb* H37Rv, respectively, was visualized at Mycobrowser website (https://mycobrowser.epfl.ch/)^17^.

### Construction of the deletion mutant and complemented strains

The unmarked deletion of *Rv0504c* (*hadD*_*Mtb*_) gene in *Mtb* H37Rv (ATCC 27294) strain was done using a previously described method^19^. Briefly, the *Rv0504c* deletion delivery vector was constructed by amplifying the upstream (0.95 kb) and downstream (1.1 kb) regions of the gene using the primer pairs EUF24 (5′-GCTGCAGCTTCTTCGACGTGGACAACA-3′) and EUR24 (5′- CAAGCTTGCCGATCAGTGTCTGGGCTTCT-3′) and EUF25 (5′-CAAGCTTGCCGAGATCCGAAGCGAAGTTA-3′) and EUR25 (5′- CGGTACCTTTCGGCTGACCCTTATTG-3′), respectively. PCR-amplified fragments were cloned into p2NIL^19^ using the restriction sites in the primers (PstI, HindIII and Kpn I, underlined in the above sequences) and the sequence was verified. The PacI cassette containing *lacZ*, *sacB*, and *hyg* from pGOAL19^19^ was introduced to construct the final vector pTACK0504G. The plasmid was electroporated into *Mtb* and single crossovers were isolated. Double crossovers were isolated from the single crossover strain as previously described^19^. Colonies were screened for the presence of the wild-type (wt) or deletion alleles by PCR using primers 0504D1 (5′-AGCCTCTAGACGCCAATCAC-3′) and 0504D2 (5′-GGCTCAAGGTTCAGCTTGTC-3′). The deletion was checked by PCR and sequencing. For complementation of *Mtb* ∆*hadD* strain, the wt copy of *Rv0504c* gene, containing its natural promoter (186 bp before the start codon), was amplified with the primer pairs 0504C1 (5′-AGCCTCTAGACGCCAATCAC-3′) and 0504C2 (5′-CAAGCTTCGTCATTGAACGGACCCTAC-3′) that incorporates a HindIII restriction site and cloned into pSC-A (Agilent). The HindIII fragment from pUC-GM-Int containing the mycobacteriophage L5 integrase, att site, and Gm resistance^40^ was inserted to make the complementing vector. The resulting plasmid pUC-*hadD*_*Mtb*_ and the empty plasmid pUC (as a control) were used to transform both *Mtb* wt and *Mtb* ∆*hadD* strains (Supplementary Table S3). For cross-complementation experiments, pUC-*hadD*_*Msm*_ plasmid carrying a wt copy of *MSMEG_0948* (*hadD*_*Msm*_) gene^16^ was used to transform *Mtb* ∆*hadD* and pUC-*hadD*_*Mtb*_ plasmid was used to transform *M*. *smegmatis* Δ*hadD* strain previously constructed^16^ (Supplementary Table S3**)**. Data were compared to those obtained for *M. smegmatis* mc^2^155 (wt) strain as well as *M. smegmatis* Δ*hadD* complemented by pUC-*hadD*_*Msm*_ and previously described^16^.

### Culture conditions and phenotyping assays

For biofilm growth and lipid analyses, *Mtb* strains were cultured as pellicles in glass bottles for 4–5 weeks at 37 °C in 7H9 broth (Difco) containing 0.2% glycerol, 10% Middlebrook ADC (Difco) and 10 µg/ml gentamycin. Cultures were inoculated with identical volumes of precultures in exponential growth phase grown in the same medium then adjusted at OD_600_ ~ 1. For the growth curves, planktonic cultures were realized under shaking (120 rpm) in the above medium supplemented with 0.05% (w/v) Tween-80, and OD_600_ was measured at different time points. For colony morphology and susceptibility to temperature assays on *Mtb* strains, liquid precultures were done in the latter medium (with Tween-80) then adjusted to the same OD and serially diluted. Five µl aliquots of each dilution were spotted on Middlebrook 7H11 medium (Sigma) supplemented with 0.5% glycerol, 10% Middlebrook OADC (Difco) and 10 µg/ml gentamycin. Cultures were incubated for at least 3 weeks at 37 °C or at 30 °C (for temperature testing). The minimum inhibitory concentrations for several antibiotics and SDS were determined in 7H9 broth supplemented with glycerol by using a colorimetric microassay based on the reduction of 3-(4,5-dimethylthiazol-2-yl)-2,5-diphenyltetrazolium bromide (MTT, Sigma-Aldrich) into formazan by metabolically active cells, as described^41^. Colony morphology and rifampicin susceptibility assays on *M. smegmatis* strains were performed as previously described^16^.

### Lipid extractions, (HP)TLC analyses and purification

The MAs and the total extractable lipids were extracted and analyzed as described previously^16^, except that the (HP)TLC plates for MAME separation were developed in ether/diethyl ether (9:1, v/v, five runs). Purification of MAMEs and compound Z was performed by preparative TLC using silica gel 60 plates (Merck) developed in the above eluent; the compounds were then scraped off and extracted from silica gel three times with diethyl ether.

### Lipid structural analyses

Peracetylation of compound Z was performed in pyridine:acetic anhydride 1:1 for 1 h at 100 °C. After drying, three extractions with H_2_O:diethyl ether (1:1) were done; the ether phases were collected, washed three times with water and dried. MALDI-TOF MS analyses were performed in the positive ionization and reflectron mode, using the 5800 MALDI-TOF/TOF Analyzer (Applied Biosystems/ABsciex) equipped with a Nd:YAG laser (349 nm wavelength) as described previously^16^. ^1^H-NMR and ^1^H-^1^H-NMR COSY spectra were recorded in CDCl_3_ at 298° K using a 600-MHz Bruker Avance III spectrometer (Bruker Biospin) equipped with a TCI cryoprobe. Chemical shift values were referenced to CHCl_3_ resonance (δH 7.26 ppm). The quantification of MAME unsaturations was performed with TopSpin 3.5 pl7 software.

### Mouse infection experiments

Virulence studies were performed in SCID mice. Briefly, groups of 6 week old C.B-17/Icr SCID mice (Charles River) were intravenously infected with a bacterial suspension containing 1–2 × 10^5^ CFU/mouse. One day and 28 days after infection, mice were euthanized, and spleen and lungs were homogenized in 2 ml tubes containing 500 µl Sauton medium and 2.5 mm diameter glass beads using an MM300 apparatus (Qiagen). CFU numbers in target organs were determined by plating 5- or 10-fold serial dilutions of organ homogenates on solid medium and incubation at 37 °C. These studies were approved by the Institut Pasteur Safety Committee (Protocol 11.245; experimentation authorization number 75–1469), in accordance with European and French guidelines (Directive 86/609/CEE and Decree 87–848 of 19 October 1987), and implicating approval from local ethical committees (CETEA 2013–0036 and CETEA dab180023).

## Supplementary information

Supplementary Information.

## Data Availability

All data generated or analysed during this study are included in this published article (and its Supplementary Information files).
