# Supplementary material for: Discovery of a novel dehydratase of the fatty acid synthase type II critical for ketomycolic acid biosynthesis and virulence of Mycobacterium tuberculosis
Source: Sci Rep. 2020 Feb 7;10:2112. doi: 10.1038/s41598-020-58967-8 (PMC7005898; doi:10.1038/s41598-020-58967-8)
Supplement: Supplementary file 1 — Supplementary Information. [file 41598_2020_58967_MOESM1_ESM.pdf]

Discovery of a novel dehydratase of the fatty acid synthase type II critical for ketomycolic acid biosynthesis and virulence of *Mycobacterium tuberculosis*

Authors: Cyril Lefebvre[1], Wafa Frigui[2], Nawel Slama[1§], Françoise Lauzeral-Vizcaino[1ψ], Patricia Constant[1], Anne Lemassu[1], Tanya Parish[3], Nathalie Eynard[1], Mamadou Daffé[1], Roland Brosch[2], Annaïk Quémard[1*]

Affiliations:
[1]Département Tuberculose & Biologie des Infections, Institut de Pharmacologie et de Biologie Structurale, UMR5089, Université de Toulouse, CNRS, UPS, 31077 Toulouse Cedex 04, France, [2]Institut Pasteur, Unit for Integrated Mycobacterial Pathogenomics, CNRS UMR3525, Paris, France. [3]TB Discovery Research, Infectious Disease Research Institute, Seattle, WA, USA.
[§]Currently at Toulouse White Biotechnology (UMS INRA / INSA / CNRS), Ramonville Saint-Agne. [ψ]Currently at Centre de Recherches en Cancérologie de Toulouse, UMR1037 Inserm/UPS, Toulouse.

**Correspondence should be addressed to A.Q. (email: annaik.quemard@ipbs.fr)*

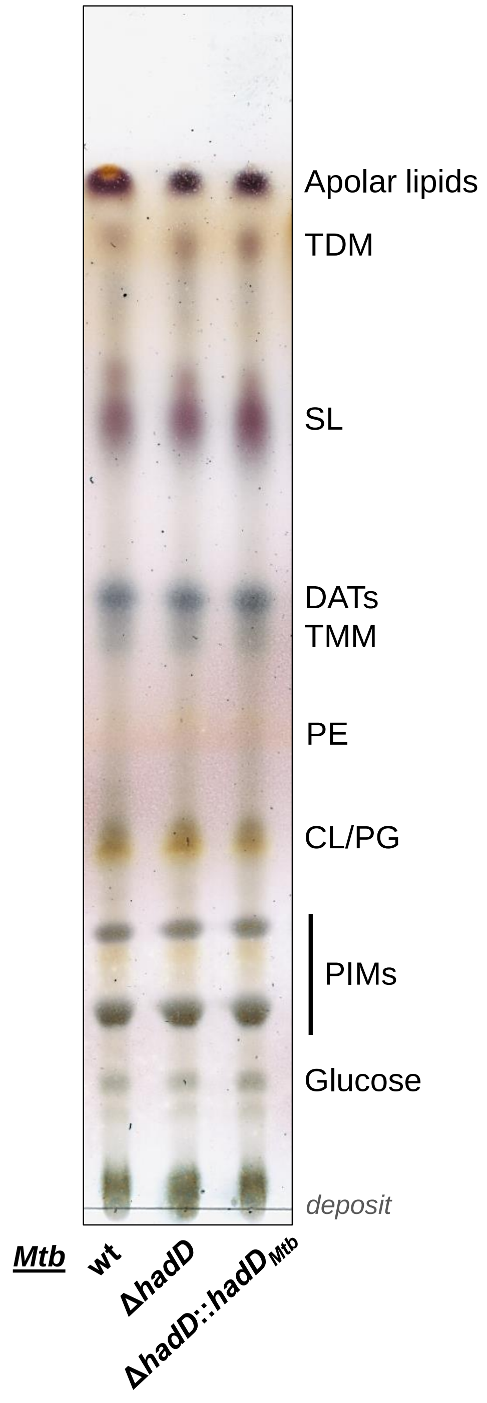

Supplementary Figure S1. The extractable lipid profile does not change in *Mtb* Δ*hadD* mutant as compared to *Mtb* wt. The thin layer is representative of at least three independent experiments. Identical amounts of total extractable lipid mixtures were loaded onto TLC plate developed in $CHCl_3$:CH_3OH:H_2O (65:25:4, v/v/v). The spots were revealed by anthrone spraying and heating. CL, cardiolipins; DATs, diacyltrehaloses; PE, phosphatidyl ethanolamine; PG, phosphatidyl glycerol; PIMs, phosphatidyl-*myo*-inositol mannosides; TDM, trehalose dimycolate; TMM, trehalose monomycolate; SL, sulfoglycolipids.

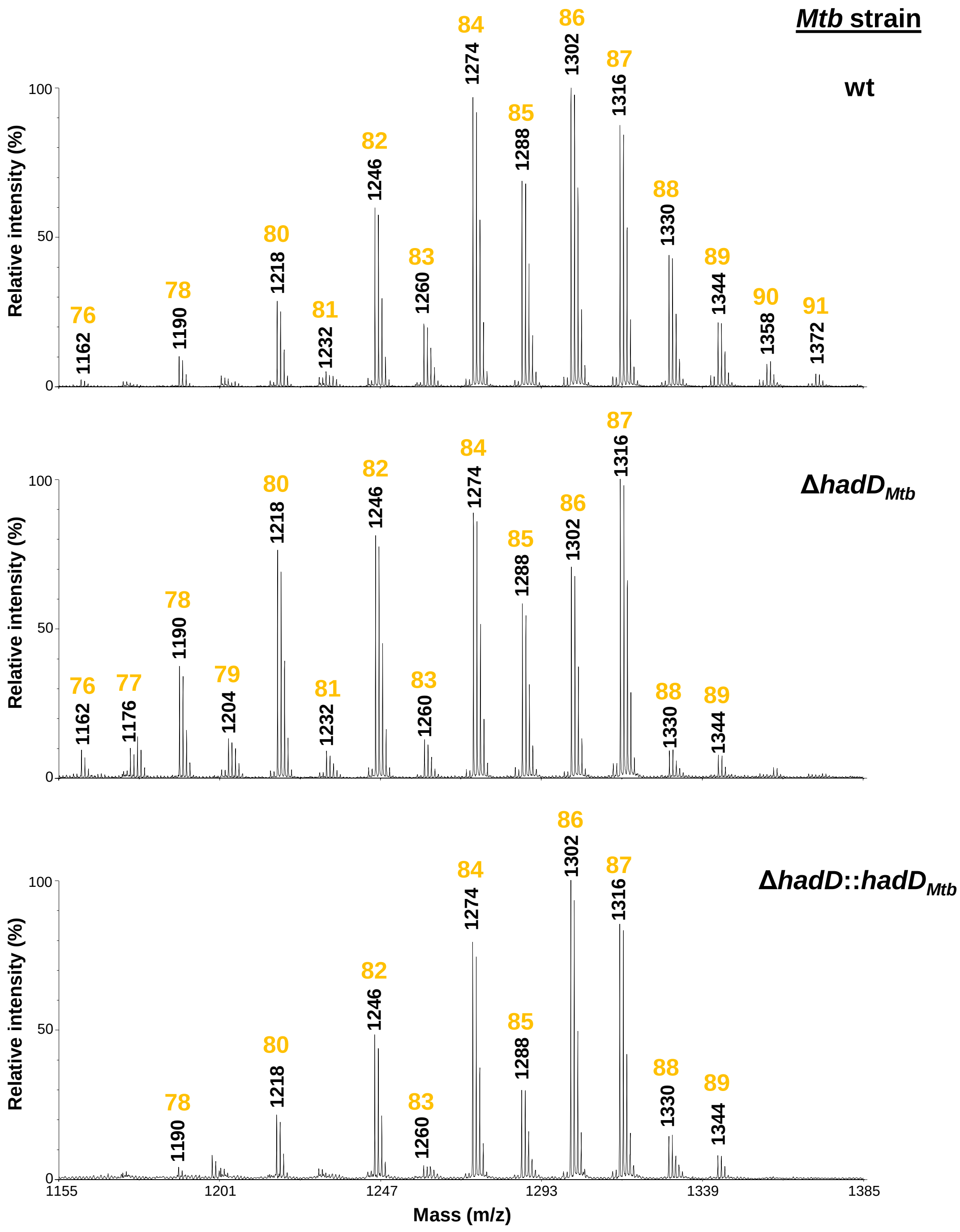

Supplementary Figure S2. HadD$_{Mtb}$ influences the size distribution of keto-mycolic acids. The relative content of the long chain keto-MAs decreases in favor of the short chain keto-MAs in *Mtb* Δ*hadD* with respect to *Mtb* wt. The wt profile is partially recovered in the complemented strain. MALDI-TOF MS spectra of the purified keto-MAMEs from the mentioned *Mtb* strains. Ion peaks are labeled with the matching total carbon number (of the free acid form). They correspond to monosodium adducts. The spectra are representative of three independent experiments.

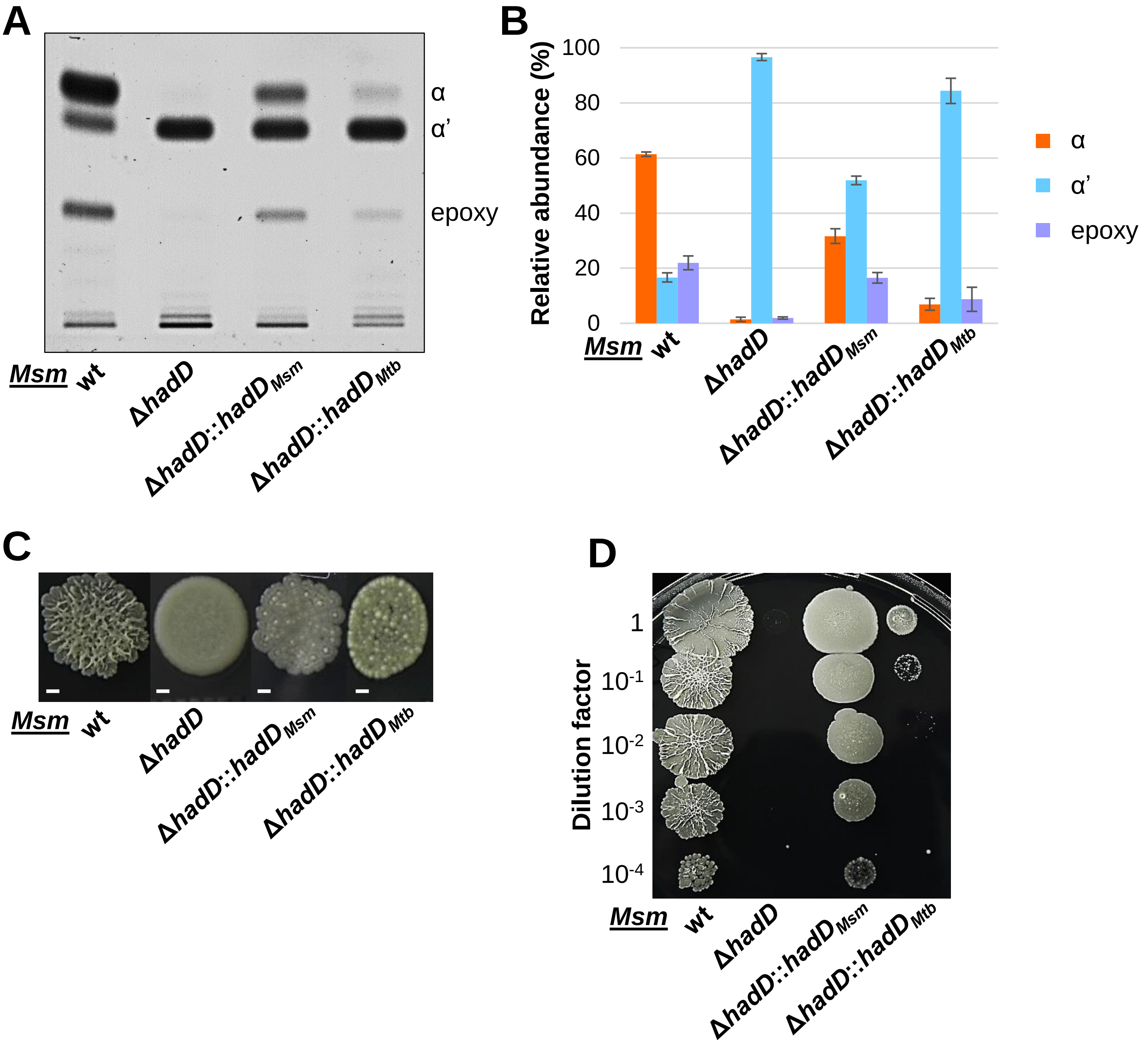

Supplementary Figure S3. HadD$_{Mtb}$ and HadD$_{Msm}$ proteins have similar functions but are not orthologs. Phenotypic analysis of the cross-complemented *M. smegmatis* Δ*hadD*::*hadD*$_{Mtb}$ strain. All of the data are representative of at least three independent experiments. (A) HPTLC profiles of MAMEs. Five µg of each MA mixture were loaded onto a HPTLC plate developed in petroleum ether/diethyl ether (9:1, v/v), and stained by immersion in $CuSO_4$ and heating. (B) MA distribution in the different strains deduced from the quantification of the HPTLC band intensities as in panel A. Data are means ± average deviations of at least three independent experiments. (C) Colony morphology of the different strains. Five µl culture aliquots at identical OD were spotted on 7H10-based medium and grown for 3 days at 37°C. Scale bars represent 1 mm. (D) Susceptibility to rifampicin. Precultures at identical OD were serially diluted then spotted on 7H10 medium supplemented with rifampicin and incubated at 37°C. Data for *M. smegmatis* wt, Δ*hadD* and Δ*hadD*::*hadD*$_{Msm}$ strains have been previously reported[1].

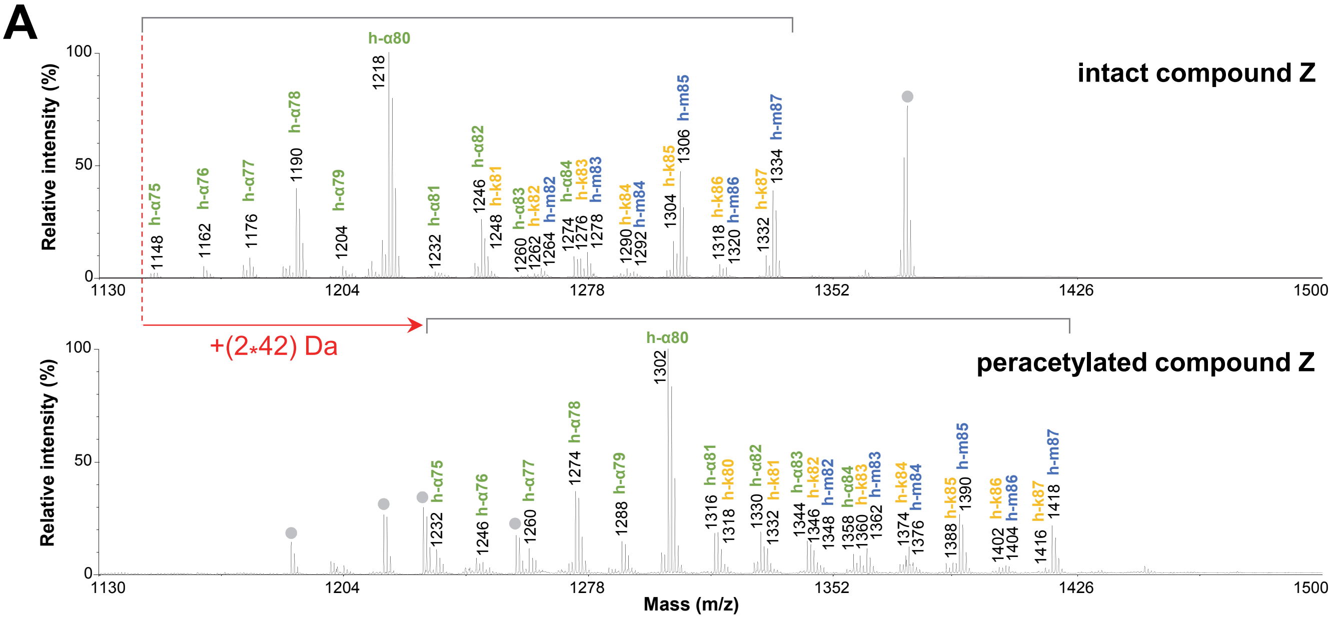

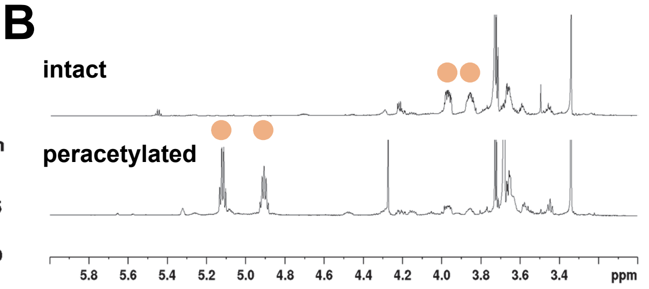

Atom	δ ¹H (ppm)	Multiplicity
2	2.69	multiplet
3	5.14	multiplet
4	1.88	multiplet
5	4.93	multiplet
6	1.55	multiplet

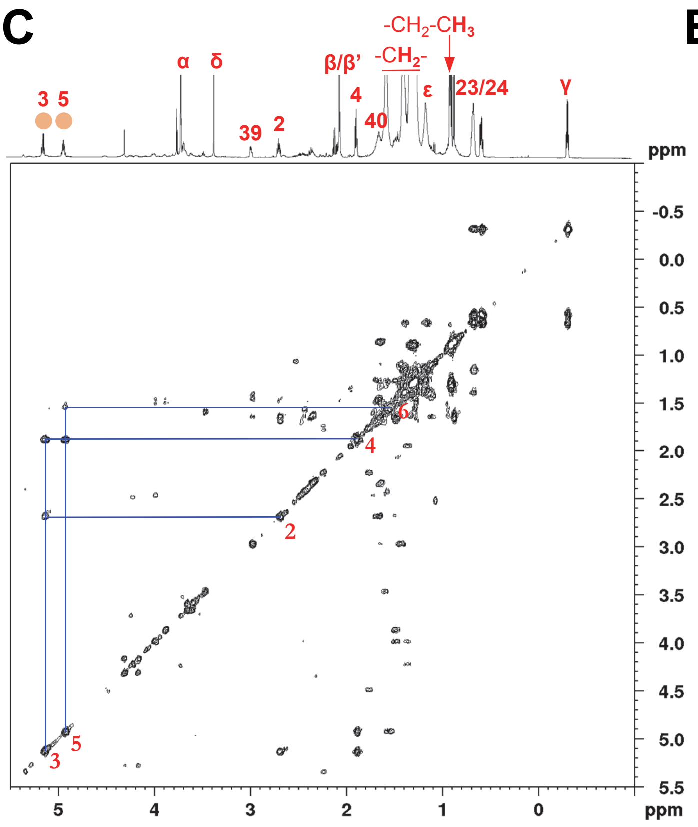

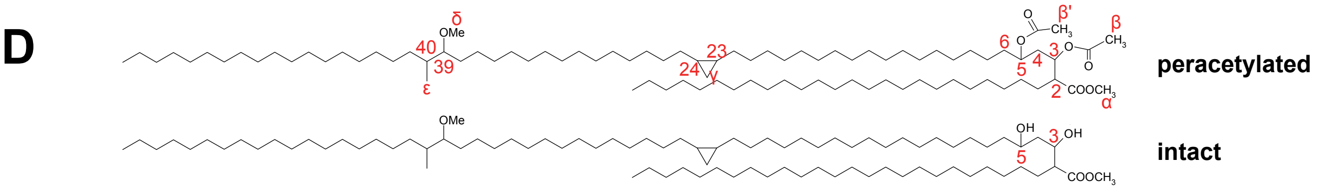

Supplementary Figure S4. *See legend next page.*

Supplementary Figure S4. Structural analysis of purified compound Z. Compound Z corresponds to a mixture of 5-hydroxylated α-, methoxy- and keto-MAs (labeled h-α, h-m and h-k, respectively). (A) MALDI-TOF MS spectra of intact compound Z (top panel) and peracetylated compound Z (bottom panel). The ion peaks are labeled with the matching MA type and the total carbon number (of the free acid form). Peaks correspond to monosodium adducts. Grey dots indicate unidentified peaks. (B) Magnification of the ^{1}H-NMR spectra of intact compound Z (top) and peracetylated compound Z (bottom). Signals given by the protons on carbons carrying a hydroxyl group (top) or a *O*-acetyl group (bottom) are labeled by orange dots. (C) ^{1}H-^{1}H-NMR COSY spectrum of peracetylated compound Z. The spin systems given by the protons on carbons carrying the *O*-acetyl groups (labeled by orange dots) are indicated. Chemical shifts and multiplicities are specified in the table. The atom numbers or Greek letters refer to the protons of peracetylated compound Z as defined in the structure in panel (D). (D) Structure of compound Z. A peracetylated (top) or intact (bottom) 5-hydroxylated methoxy-MAME was drawn, as an example.

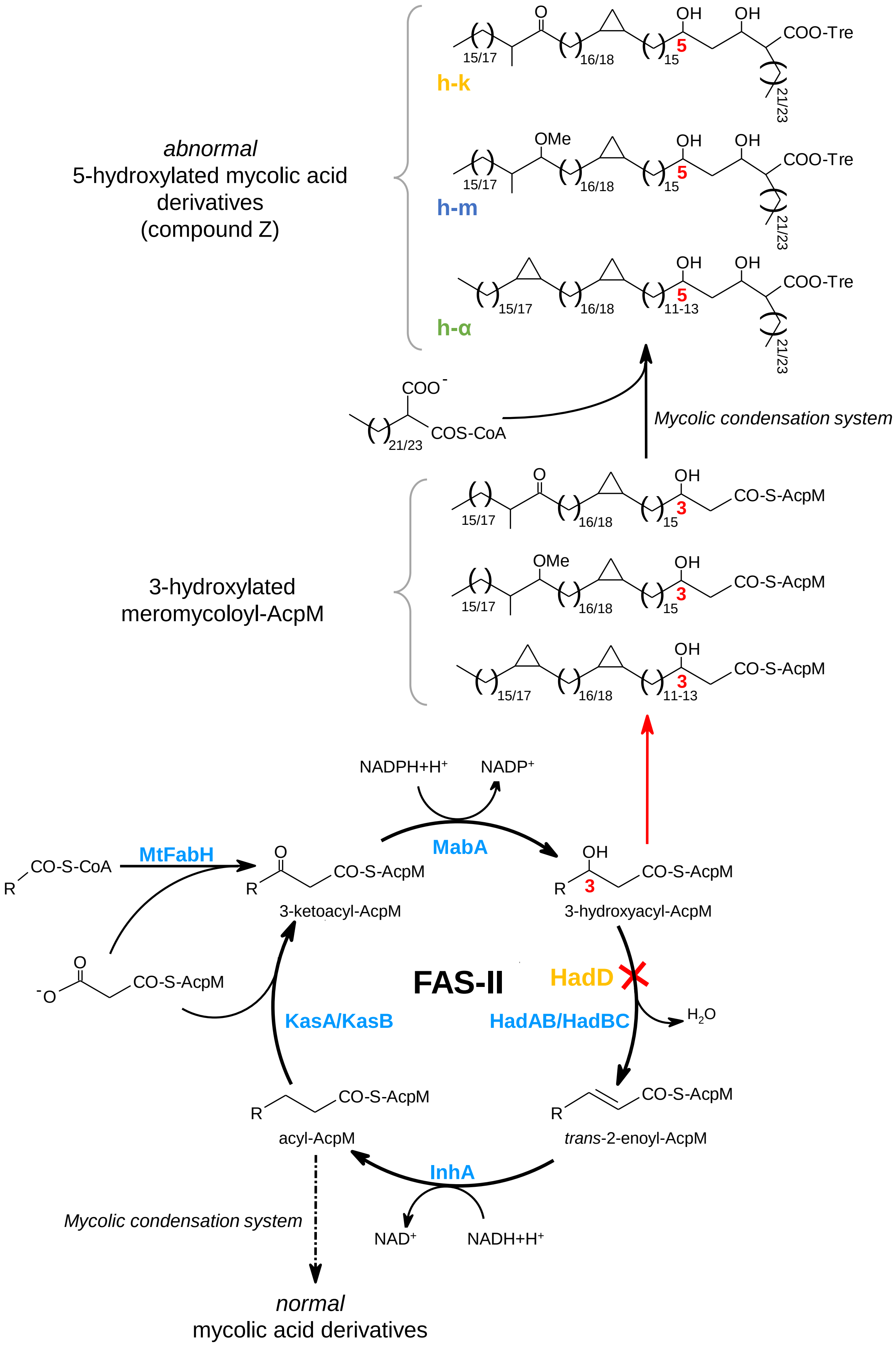

Supplementary Figure S5. *See legend next page.*

Supplementary Figure S5. The accumulation of FAS-II intermediates triggered by *hadD*$_{Mtb}$ inactivation leads to the biosynthesis of abnormal MAs. In the absence of HadD$_{Mtb}$ protein, the dehydratation step of the last FAS-II elongation cycles within the oxygenated MA pathways (see Fig. 6) is most likely blocked (red cross), preventing the formation of mature meromycoloyl chains. As a consequence, 3-hydroxylated meromycoloyl-AcpM intermediates, corresponding to HadD$_{Mtb}$ substrates, accumulate (red arrow), then are taken over by the mycolic condensation system, leading to the synthesis of abnormal 5-hydroxylated MAs (compound Z). The 5-hydroxylated α-, methoxy- and keto-MA chains are labeled by h-α, h-m and h-k, respectively, as in Fig. S4 (panel A). The position of the additional hydroxy group is indicated in red. These molecules are *cis*- or *trans*-cyclopropanated; for better clarity, only *cis*-cyclopropanated molecules were drawn. AcpM, mycobacterial FAS-II acyl carrier protein.

Mtb strain	Minimal inhibitory concentration[a]				
	**Rifampicin (µg/ml)**	**Isoniazid (µg/ml)**	**Ethambutol (µg/ml)**	**Ciprofloxacin (µg/ml)**	**SDS (%)**
wt	0.06	0.08	12.5	0.83	0.003
Δ*hadD*	0.016	0.04	6.25	0.83	0.003
Δ*hadD*::$hadD_{Mtb}$	0.03	0.04	6.25	0.83	0.006

[a]MIC values are representative of data from at least three independent experiments. They were measured by MTT assays and determined as the lowest concentration of compound yielding 99.9% inhibition of the bacterial growth.

Supplementary Table S1. Impact of *hadD* deletion on the sensitivity of *M. tuberculosis* to antibiotics and SDS.

MAME	chemical shifts (ppm)	*Mtb* wt			*Mtb* Δ*hadD*				*Mtb* Δ*hadD*::*hadD*$_{Mtb}$			*Mtb* Δ*hadD*::*hadD*$_{Msm}$		
		α	methoxy	keto	α	methoxy	keto	Z	α	methoxy	keto	α	methoxy	keto
cyclopropane #/molecule[a]		2.02	0.95	1.03	2.03	1.02	0.95	1.65	2.08	1.04	1.01	2.05	1.00	0.97
cis (%)[b]	-0.335<br>0.557<br>0.642	100	100	83	100	100	67	99	100	95	77	100	98	82
trans (%)[b]	0.115<br>0.181<br>0.444	-	-	17	-	-	33	1	-	5	23	-	2	18

[a]The number of cyclopropanes per molecule (degree of unsaturation) was determined by integration of the NMR signal areas, using the three protons of the methyl ester function of MAMEs as an internal standard. In the standard MAs (as those from *Mtb* wt), the degree of unsaturation is equivalent to 2 for α-MAs and to 1 for methoxy- and keto-MAs. No double bonds were detected in these MAMEs. [b]Relative distribution of *cis* and *trans* cyclopropanes.

Supplementary Table S2. ^{1}H-NMR analysis of the unsaturations in purified MAs of the different *Mtb* strains

Bacterial strain	Description	Source
Mtb wt::pUC	Wild type *Mtb* H37Rv (ATCC 27294) strain transformed by the empty integrative vector pUC-Gm-Int (carrying a Gm^R cassette)[a]	This work
Mtb Δ*hadD*::pUC	*Mtb* H37Rv with a 308 bp unmarked in-frame deletion in *Rv0504c* (*hadD*$_{Mtb}$) gene transformed by the empty vector pUC-Gm-Int	This work
Mtb Δ*hadD*::pUC-*hadD*$_{Mtb}$	*Mtb* Δ*hadD* complemented with vector pUC-Gm-Int carrying a wt copy of *Rv0504c* gene downstream its natural promoter	This work
Mtb Δ*hadD*::pUC-*hadD*$_{Msm}$	*Mtb* Δ*hadD* transformed by vector pUC-Gm-Int carrying a wt copy of *MSMEG_0948* (*hadD*$_{Msm)}$ gene downstream its natural promoter	This work
Mtb wt::pUC-*hadD*$_{Mtb}$	*Mtb* H37Rv wt strain transformed by vector pUC-Gm-Int carrying a wt copy of *Rv0504c* gene downstream its natural promoter	This work
Msm wt::pUC[b]	Wild type *Msm* mc²155 strain transformed by the empty integrative vector pUC-Gm-Int (carrying a Gm^R cassette)	Ref. 1
Msm Δ*hadD*::pUC	*Msm* mc²155 where *MSMEG_0948* (*hadD*$_{Msm}$) gene has been completely replaced by a streptomycin resistance cassette, and transformed by the vector pUC-Gm-Int	Ref. 1
Msm Δ*hadD*::pUC-*hadD*$_{Msm}$	*Msm* Δ*hadD* complemented with vector pUC-Gm-Int carrying a wt copy of *MSMEG_0948* gene downstream its natural promoter	Ref. 1
Msm Δ*hadD*::pUC-*hadD*$_{Mtb}$	*Msm* Δ*hadD*$_{Msm}$ transformed by vector pUC-Gm-Int carrying a wt copy of *Rv0504c* (*hadD*$_{Mtb)}$ gene downstream its natural promoter	This work

[a]Gm^R refers to gentamycin resistance. [b]*Mms*, *M. smegmatis*.

Supplementary Table S3. Strains and plasmids used in the present work.

REFERENCES

1 Lefebvre, C. *et al*. HadD, a novel fatty acid synthase type II protein, is essential for alpha- and epoxy-mycolic acid biosynthesis and mycobacterial fitness. *Scientific Reports* **8**, 6034, doi:10.1038/s41598-018-24380-5 (2018).
